# Molecular diagnosis of *Toxoplasma gondii* infection in Libya

**DOI:** 10.1186/s12879-016-1491-5

**Published:** 2016-04-16

**Authors:** Aisha Gashout, Ahmad Amro, Mabruk Erhuma, Hamida Al-Dwibe, Eanas Elmaihub, Hamouda Babba, Nabil Nattah, Abdalhafid Abudher

**Affiliations:** Faculty of Medical Technology Pathology Department, University of Tripoli, Tripoli, Libya; Faculty of Pharmacy, Al-Quds University, Main Campus, Abu Dis, P.O. Box 5100, Jerusalem, Palestine; Medical Laboratory Department, Immunology Unit, Tripoli Central Hospital, Tripoli, Libya; Faculty of Medicine, Dermatology Department, University of Tripoli, Tripoli, Libya; Scientific College – Sabrata, Zoology Department, University of Zawia, Zawia, Libya; Laboratoire de Parasitologie-Mycologie à la Faculté de Pharmacie, Monastir, Tunisia; Genetic Laboratory at Bio- technologies Researches Centre, Tripoli, Libya; Libyan National Centre for Disease Control, Tripoli, Libya

**Keywords:** Libya, *Toxoplasma*, PCR, Semi nested PCR, IgM, IgG, ELISA

## Abstract

**Background:**

*Toxoplasma gondii* infections are prevalent in humans and animals throughout Libya. Current diagnosis is based on detection of *Toxoplasma*-specific IgM and IgG. In this study, we established and optimized a diagnostic PCR assay for molecular diagnosis of *T. gondii* in Libya.

**Methods:**

From January to December, 2010, 177 blood and serum samples were collected from suspected patients. This includes: 140 women who have had spontaneous abortions, 26 HIV-positive patients, nine patients with leukemia and lymphoma, and two infants with ocular infection. Samples were screened for anti-*Toxoplasma* IgG and IgM antibodies before DNA extraction. The surface antigen gene 2 (SAG2) was targeted in a semi-nested PCR to amplify a 999 bp and a 614 bp fragment in the first and the second run respectively.

**Results:**

A total of 54/140 (38.5 %) women who have had spontaneous abortions, 23/26 (88 %) HIV patients, 6/9 (66.6 %) of the leukaemia and lymphoma patients, and one child with ocular infection were seropositive for anti-*Toxoplasma* IgG and/or IgM. Genomic DNA was extracted from 38 selected seropositive samples. The PCR was sensitive enough to detect DNA concentration of 12 ng/μL. PCR analysis was performed for 38 selected seropositive patients (16 women who have had spontaneous abortions, 15 positive HIV patients, six leukaemia patients and one child with ocular infection). Our designed primers were successfully amplified in 22/38 (57.9 %) samples; 5/12 (35.7 %) from serum and 17/26 (65.8 %) from whole blood samples. All PCR positive samples were IgG-positive except two samples which were IgM and IgG & IgM-positive serum samples respectively. The semi-nested PCR confirmed five more samples. These included two leukaemia and two HIV-positive whole blood samples and one serum sample from an aborted woman.

**Conclusion:**

The ability of PCR to diagnose active toxoplasmosis is needed in immunocompromised patients and congenital toxoplasmosis cases, especially when serological techniques fail. For the first time in Libya, we established and optimized semi-nested PCR of SAG2 gene. The developed PCR method was able to detect as little as 12 ng/μL of *T. gondii* DNA and was useful to diagnose the diseases in women who have had spontaneous abortions, HIV-positive patients, patients with leukemia and lymphoma, and infants with ocular infection.

## Background

*Toxoplasma gondii* infections are prevalent in humans and animals worldwide. It has been estimated that one-third of the world population has been exposed to this parasite [1, 2]. The infection is acquired by ingesting tissue cysts from undercooked or raw meat, consuming food or drink contaminated with oocysts shed by felids, or by accidentally ingesting oocysts from the environment [3].

Although the course of the primary infection is usually subclinical and the vast majority of infected human populations remain asymptomatic, the infection can cause significant morbidity and mortality in certain groups. This includes encephalitis, chorioretinitis, congenital infection and neonatal mortality [4]. Transmission to the fetus occurs in women who acquire their primary infection during gestation and can result in visual and hearing loss, mental and psychomotor retardation, seizures, hematological abnormalities, hepatosplenomegaly, or death [5]. The global annual incidence of congenital toxoplasmosis was estimated to be 190,100 cases [6]. High burdens of congenital toxoplasmosis, which where estimated as the highest among all food-borne pathogens [7], were seen in South America and in some Middle Eastern and low-income countries [6]. Moreover, toxoplasmic encephalitis due to reactivation of latent tissue cysts is the most common clinical presentation of toxoplasmosis among persons with AIDS [8–11]. The infection is typically observed in the later stages of human immunodeficiency virus (HIV) infection, when persons become severely immunosuppressed [12, 13]. The incidence of encephalitis in AIDS patients in the general population is directly related to the prevalence of anti-*T. gondii* antibodies [8]. Global seroprevalence of toxoplasmosis is continuingly evolving, subject to regional socioeconomic parameters and population habits. It presents in every country in the world and seropositivity rates range from less than 10 % to over 90 % [14].

In African countries, numerous studies performed in the early 1990s with limited follow-up exists even for the general population of these countries. Recent review by Pappas et al. [14] summarized prevalence rates in Egypt (57.9 %), Tunisia (58.4 %), Morocco (50.6 %), Nigeria (20.8 %), Mali (21 %), Benin (3.6 %), Gabon (71.2 %), Madagascar (83.5 %), and Senegal (40.2 %). However, limited studies about Libyan toxoplasmosis are available. Seroprevalence of toxoplasmosis among Libyan pregnant women in Benghazi ranges between (44.8 %) [15], (47.4 %) [16], and (50 %) [17], and among non-pregnant Libyan women in Tripoli was estimated to be around (18.14 %) [18], (43.4 %) among adult males, and (43.7 %) of school children [19]. Gashout et al. has shown that (17.6 %) of women who suffered from spontaneous abortion in Tripoli were seropositive for toxoplasmosis [20]. Moreover, prevalence of congenital toxoplasmosis was found to be (44 %) in Tripoli [21]. However, no comprehensive epidemiological analysis was done at the national level in Libya. All previous studies were based on detection of *Toxoplasma*-specific IgM and IgG. Specific and sensitive molecular diagnostic tools have not yet been implemented and information about disease distribution, parasite life cycle, and combining risk factors is limited.

Due to their high sensitivity and specificity, molecular methods are now recognized as an essential diagnostic tool for maternal and congenital toxoplasmosis [22–30], toxoplasmosis in immunocompromised individuals [31, 32], and ocular toxoplasmosis [33–35]. In most developing countries, including Libya, these methods are not widely used in clinical settings for routine diagnosis and therapeutic management, as they are expensive and time consuming techniques. Current diagnosis of toxoplasmosis in Libya is based on serological methods which have varied sensitivity and specificity depending on the test used [36, 37]. Moreover, serological tests may fail to detect *T. gondii* infection in certain immunocompromised patients due to the fact that the titres of specific anti-*Toxoplasma* antibodies may fail to rise at the time of diagnosis [38–40]. Hence direct observation of the parasite in biological samples by Polymerase Chain Reaction (PCR) is a major breakthrough for the diagnosis and management of toxoplasmosis [41].

In this study, we described establishment, optimization, and application of diagnostic PCR assay to amplify SAG2 gene of *T. gondii* from Libyan HIV patients, women who have had spontaneous abortions, leukaemia, and ocular infection patients who were sero-positive for specific *Toxoplasma* antibodies. To the best of our knowledge, this is the first molecular study of human toxoplasmosis in Libya focusing on establishment of molecular diagnostic technique.

## Methods

### Patients and samples

A total of 177 blood and serum samples were collected from clinically diagnosed patients during January-December 2010. This includes; 140 women who have had spontaneous abortions from out-patient departments, 26 HIV-positive patients from infectious department in Tripoli Central Hospital, nine patients from African Oncology Institute in Sabrata diagnosed with leukemia and lymphoma, and two infants with ocular infection from private Alsharkh Laboratories in Zawia City. Patient’s data including, age, sex and location, were collected for epidemiological analysis.

### Serologic tests

Approximately 5 ml of venous blood were collected from each patient. Two ml were added to plain tube to get serum. The rest was transferred into tube with EDTA for DNA extraction. Serum was separated from the whole blood by centrifugation at 3000 rpm for 5 min and screened for anti-*Toxoplasma* IgG and IgM antibodies by using standard ELISA commercial kits (Human Gesellschaft für Biochemica und Diagnostica Gmb*H*, Wiesbaden, Germany) in accordance with the manufacturer’s instruction. Moreover, the Architect Toxo IgG and Toxo IgM assays (Abbott Laboratories, Wiesbaden, Germany) was used to confirm ELISA results for a subset of patients to avoid false negatives.

### Parasites preparation and genomic DNA extraction

Reference strain RH (type I) was used as positive control. A stock solution of 1000 tachyzoites/100 μl of PBS was prepared and kept at − 80 °C until used. The genomic DNA was extracted from the RH *T. gondii* tachyzoites, blood samples spiked with different concentrations of tachyzoites, and from patient’s serum and whole blood samples using PureLink™ Genomic DNA Kit for purification of genomic DNA (Invitrogen) following the manufacturer’s instructions. Briefly, cells were lysed and digested with 20 μl of Proteinase K, RNase A (50 Mm Tris-HCl, Ph 8.0,10 Mm EDTA) and 200 μl of Lysis/Binding Buffer at 55 °C for 10 min. Absolute ethanol (200 μl) was added and the mixture was transferred to the PureLink™ Spin column in a 2 ml collection tube and centrifuged for 1 min. The columns were washed twice, and the DNA was eluted from the columns with 50 μl of elution buffer (10 mM Tris-HCl, pH 9.0, 0.1 mM EDTA). Spectrophotometric analysis was applied to measure DNA concentration and purity.

### Polymerase chain reactions (PCR) and semi-nested PCR

The surface antigen gene 2 (SAG2), which encode the tachyzoite surface proteins p22 was targeted as described elsewhere [42–45] with modifications. Briefly, a fragment of SAG2 gene (Gene bank: AF 24969) [46] was amplified using two primers; forward (TOXO 29) and reverse (TOXO 1027) to produce a 999 bp fragment (Table 1). Semi-nested PCR was then performed to confirm specificity of first round products by using forward (TOXO 409) and reverse (TOXO 1027) to produce a 614 bp fragment (Table 1). We designed all primers by using online Primer3 Output; http://primer3.ut.ee/.

**Table 1:**
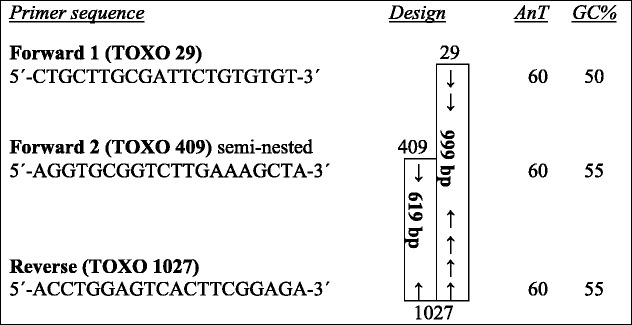
Design of SAG2 PCR and semi-nested PCR analysis

*AnT* Annealing temperature

Conventional PCR was optimized in a 50 μL reaction that includes; 5 μL of High Fidelity 10× PCR buffer (20 mM Tris-HCl, pH 7.5, 100 mM KCl, 1 mM Dithiothreitol (DTT), 0.1 mM EDTA, 0.5 % Tween 20, 0.5 % Nonidet P40, 50 % glycerol), 4 μL of (25 mM) MgCl2, 5 μL of (10 mM) dNTPs, 0.5 μL of each (50 pmol) primer, 0.25 μL of (5U/μl) High Fidelity enzyme mix and 10 μL of DNA template, and 29.25 μL of ultrapure water. The mixture was briefly spun and placed in thermal cycler (GenAmp® PCR system, Applied Biosystem). The reaction conditions were optimized using number of modifications; temperature for primer annealing (58–60 °C), MgCl2 concentration (1.5, 2, 3, 4 mM), enzyme mix (1.1, 1.25 and 2.75U/μl) and primer concentration (12.5 16.5, 25, 41, and 45 μM).

PCR sensitivity was assessed by using serial dilutions of *T. gondii* DNA (RH strain) ranging from 12.5, 25, 50 and 100 ng/μl input per 50 μl PCR reaction as described previously [45]. The dilutions were tested by PCR to determine the minimum DNA concentration per reaction that could be detected by this protocol. Moreover, PCR sensitivity was tested by mixing 200 μl of whole blood with different concentrations of *T. gondii* tachyzoites of the RH strain (10, 100 and 1000 parasites) prior to extraction according to Howe et al. [44].

For semi-nested PCR, we took 5 μL of a 1:10 diluted PCR amplicon from the first-round as a template. The protocol for reaction conditions was used as described for first round PCR amplification. Each amplification assay contained a negative control (negative sample for IgG and IgM *T. gondii* antibodies and/or ultrapure water) and one positive control (2 μL DNA from RH *T. gondii* tachyzoites). To guarantee the reliability of the results and detect any possible contamination, all samples were processed in duplicate. The test result was considered positive if the amplified DNA fragment was clearly visible in both samples.

For DNA detection, 5 μL of amplicons were analyzed on 1.7 % agarose gel by electrophoresis and visualized by UV light and then photographed under an ultraviolet transluminator. 100 bp plus DNA Ladder (Bioron) was used as a marker. Gel quantification analysis of the bands amplified from blood samples spiked with different concentrations of tachyzoites was carried out using ImageJ 1.46r software (National Institutes of Health, Bethesda, MA, USA).

### Ethical considerations

All aspects of the study were revised and approved by the ethics committee of the Libyan National Centre for Disease and Control. Confidentiality was ensured through secure data management and no personal identifiers were in the computer system. Data and samples were labeled with anonymous identification numbers. Informed written consent was obtained from all participants. Since two study participants were infants, parents/guardians provided consent on their behalf. Test results were confidentially disclosed to the subjects following post-test counseling.

## Results

### ELISA results for *T. gondii* IgM and IgG antibodies

One hundred and forty women who have had spontaneous abortions, median age 27 year (range19–41 year) in the first 16 weeks of gestation, were clinically diagnosed and tested for *Toxoplasma* infection with ELISA. The mean length of pregnancy was 10 weeks (range 1–16 weeks) at the time of abortion and sample collection.

A total of 54/140 (38.5 %) women who have had spontaneous abortions were seropositive; 36 (66.6 %) were positive for IgG antibodies, 12 (22.2 %) for IgG and IgM antibodies, and 6 (11.1 %) for IgM. Moreover, 4 of the IgG-positive women had a history of repeated abortion.

Twenty six HIV positive patients (14 male and 12 female, mean age 37 years old) were tested for *T.gondii* infection. The HIV-positive patients were considered seropositive if the ELISA result of serum samples had detectable specific *Toxoplasma* IgG antibodies with titer ≥ 10 IU/ml. A total of 23/26 (88 %) patients were IgG positive among which 17 (74 %) had a CD4 cell-count less than 100 cells/μl of blood. CD4 levels in HIV-positive patients with seropositive toxoplasmosis (range, 3 to 273 CD4 cells/μl of blood) were lower than seronegative patients (range, 345 to 463 CD4 cells/μl of blood).

However, 6/9 (66.6 %) of the leukaemia and lymphoma patients (five males and four females, mean age 36 year), and one child (male, 4 months) with ocular infection were IgG positive (Table 2). All samples retested with Architect Toxo IgG and Toxo IgM assays confirmed the ELISA results without any discrepancies.

**Table 2:** Serological and corresponding PCR results

Groups	Serology Results				PCR and semi-nested PCR Results		
	No. of Samples	IgG	IgG&IgM	IgM	No. of Samples	Serum	Whole Blood
Women who have had spontaneous abortions	140	36	12	6	16	2/6	6/10
HIV	26	23	-	-	15	3/6	6/9
Leukaemia and lymphoma patients	9	6	-	-	6	-	4/6
Ocular infection	2	1	-	-	1	-	1/1
Total	177	66	12	6	38	5/12	17/26

### Genomic DNA Extraction and PCR optimization

**Fig. 1:**
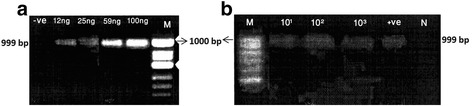
**a**: PCR amplification performed with various DNA concentrations. The quantity of DNA is shown above each lane, minimum amount detected was 12 ng. ve + =positive control. **b:** PCR for detection of *T. gondii* DNA in whole blood samples spiked with 10^1^, 10^2^ and 10^3^ tachyzoites. Lane *N* = whole blood without tachyzoites, +ve = positive control (DNA extracted from pure tachyzoites). The bands were quantified by using the software ImageJ. The percentage of the area under the curve was 10^1^ = 12 %, 10^2^ = 25 %, 10^3^ = 30 % and + ve =33 %. -ve = negative control, *M* = 100-bp DNA ladder (BIORON)

Genomic DNA was extracted from 38 selected seropositive and clinically diagnosed toxoplasmosis patients. The DNA concentration ranged from 16 to 350 ng/μl. Optimum PCR cycling parameters were: 94 °C for 4 min, then 35 cycles at 94 °C for 1 min, 58 °C for 1 min and 72 °C for 2 min. The PCR was completed with 7 min at 72 °C. Under these conditions, the PCR was sensitive enough to detect DNA concentration of 12 ng/μL of extracted DNA from purified parasite (Fig. 1a). Analytic sensitivity of 10, 100, and 1000 tachyzoites in 200 μl whole blood was predicted. We did the PCR for each concentration in triplicate and run a gel for all PCR products together, then we repeated the PCR once again to obtain a gel photo (Fig. 1b). Wile no product was detected from whole blood sample where no parasites were added (Fig. 1b).

### PCR amplification of SAG2 locus

PCR analysis was performed for 38 selected patients (16 women who have had spontaneous abortions, 15 positive HIV patients, six leukaemia patients, and one child with ocular infection). All selected cases were strong sero-positive; these included 33 samples that were positive only for IgG antibodies, four samples that were positive for both IgM and IgG antibodies, and one sample were positive for IgM.

Amplification of the SAG2 gene with our designed primers was successful in 22/38 (57.9 %) samples; 5/12 (35.7 %) from serum and 17/26 (65.8 %) from whole blood samples. All PCR positive samples was IgG-positive except two samples; one IgM-positive and one IgG and IgM-positive serum samples from women who have had spontaneous abortions. Table 2 summarizes serological and corresponding PCR results.

**Fig. 2:**
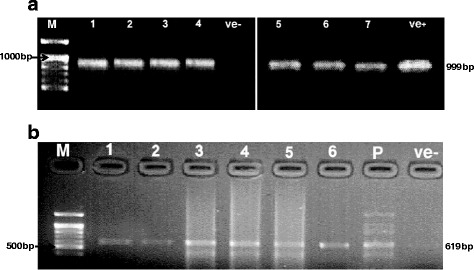
**a:** PCR amplification of *T. gondii* SAG2 gene in 1.7 % agarose gel. Lane 1 and 2 = positive HIV whole blood samples. Lanes 3 and 4 = leukaemia whole blood samples. Lane 5 and 6 = aborted woman serum and whole blood respectively. Lane 7 = patient with ocular infection. Ve + = positive control (RH strain). **b:** Semi-nested PCR amplification of *T. gondii* SAG2 gene for samples with faint band in the first PCR run. Lanes; 1 and 2 = positive HIV whole blood samples. Lanes 3 and 5 = leukaemia whole blood samples. Lane 4; aborted woman serum sample. Lane 6 = seronegative toxoplasmosis aborted woman whole blood sample. Lane *P* = positive control (RH strain). Ve- = is negative control (blanked water), *M* = 100 bp DNA Ladder (BIORON)

Figure 2a shows the PCR products resulted from the first round PCR and the semi-nested PCR which were 999 bp and 619 bp, respectively. Semi-nested PCR was done for PCR products which gave faint bands in the first round. By this PCR we confirmed five more samples. These included two leukaemia and two positive HIV whole blood samples, and one aborted woman serum sample (Fig. 2b).

## Discussion

PCR has been consistently used to detect DNA of *T. gondii* in various biological samples and has showed higher sensitivity in diagnosis compared to serological tests and culture [25, 47–50]. Moreover, the potential of PCR to diagnose active toxoplasmosis is of great importance for immunocompromised patients and congenital toxoplasmosis particularly when serological techniques failed [51]. Most researchers have used the B1 and SAG1 for detection of *Toxoplasma* [43, 52–54]. However, a nested PCR assay based on the polymorphic SAG2 locus was developed [43, 44]. SAG2 gene encodes two separate forms of the surface tachyzoite protein p22 that are recognized by strain-specific monoclonal antibodies and allow for adequate genotyping of *T. gondii* [44, 55]. The genetic analysis based on this gene requires a small amount of DNA, thus allowing it to be amplified directly from clinical samples [44].

For the very first time in Libya, we developed a sensitive and specific PCR of SAG2 gene to detect *T. gondii* in clinical samples. Positive results were detectable after the first round of PCR. However, semi-nested PCR was essential to confirm specificity of first round products with faint band intensity. This investigation was based on the combined results of positive serological tests for IgG and/or IgM antibodies, which confirms *T. gondii* DNA in whole blood and serum samples among different patients (women with history of repeated abortion, positive HIV patients, leukaemia and congenital cases).

As for all parasitic diseases, the PCR diagnosis of toxoplasmosis is not standardized [41]. Therefore, we adjusted PCR conditions to give optimum sensitivity and specificity without appearance of artifacts. The use of this assay allowed for a highly sensitive detection of less than 10 tachyzoites of *T. gondii* DNA and a minimum concentration of 12 ng/ml. The detection limit of the conventional PCR varied depending on the amounts of pure *T. gondii* tachyzoites that were mixed with whole blood. A decreased performance of conventional PCR may be expected when exceeding certain amount of non-specific DNA in a reaction volume. Interestingly, the conventional single step PCR assay targeting SAG2 in the first round was successful as in the previous studies used a nested PCR protocol targeting the same gene [44, 55]. Moreover, the semi-nested PCR system may increase the sensitivity due to its dilution effect between the first and the second round of PCR if inhibitory factors are present. However, the risk of carry-over contamination between first and second rounds of PCR should be considered to avoid false positive results which may make this approach less suitable in the routine laboratory tests.

In the present study, discrepancies between serology and PCR results were detected since 22/38 (57.9 %) of tested seropositive samples were PCR positive. Possible explanation is that the clearance time for *Toxoplasma* DNA from the patient’s blood was estimated to be 5.5–13 weeks [56]. Based on this, the presence of *Toxoplasma* DNA in the peripheral blood probably indicates a recent infection or apparent parasitaemia that is likely to be clinically significant. Conversely, a small number of parasites might have been released from tissue into the blood at a subclinical level and their presence can be detected only by PCR [57].

In this study, *T. gondii* DNA was detected only in 2/5 IgM positive women who have had spontaneous abortions and 3/11 women showed no evidence of infection by PCR, though IgG antibodies were detected. This can be attributed to the presence of a long standing immunity to toxoplasmosis or cross-reactive antibodies [58, 59] and confirm the sensitivity and specificity of PCR analysis for detecting recent infection in early pregnancy [60]. This is in agreement with previous reports that PCR is recommended over serologic techniques for diagnosis of toxoplasmosis [61–63].

*Toxoplasma* DNA was detected in one of two children clinically diagnosed with ocular infection. The first child was both PCR and seropositive at 4 months of age. The second child was both PCR and seronegative at 8 years of age. This indicated that our PCR assay may be used to confirm ocular toxoplasmosis and to differentiate it from other ocular diseases. Our results are in agreement with previously published reports [64].

The emergence of human immunodeficiency virus HIV in Libya has increased the need for more sensitive and reliable diagnostic methods to diagnose opportunistic infections like toxoplasmosis. Latent *T. gondii* infection in 30–50 % of HIV patients have a high risk of progressing to toxoplasmic encephalitis [65, 66]. In our study, the *T.gondii* DNA was detected in (60 %) of the seropositive toxoplasmosis HIV/AIDS patients with CD4 count less than 100 cells/μl. Moreover, the average CD8 count was significantly higher for patients who had negative PCR result (data not shown). Serological tests are limited with delayed or impaired production of antibodies in immunocompromised patients. Hence, PCR approaches are superior for diagnosing such cases.

## Conclusion

Diagnosis of *T. gondii* infection in Libya is based on serological detection of specific anti- *Toxoplasma* immunoglobulin, which has varied sensitivity and specificity, may fail to detect infection especially in immunocompromised patients. For the first time in Libya, we established and optimized semi-nested PCR of SAG2 gene, which is a reliable diagnostic technique with adequate sensitivity and specificity when used to detect *T. gondii* DNA in different clinical settings. The developed PCR method was able to detect as little as 12 ng/μL of *T. gondii* DNA and was useful to diagnose the disease in women who have had spontaneous abortions, HIV-positive patients, patients with leukemia and lymphoma, and infants with ocular infection.

### Ethics approval and consent to participate

All aspects of the study were revised and approved by the ethics committee of the Libyan National Centre for Disease and Control. Informed written consent was obtained from all participants or their parents/guardians.

### Consent for publication

Not applicable.

### Availability of data and materials

All the data is contained within the manuscript.

## Abbreviations

ELISA: enzyme-linked immunosorbent assay
Ig: immunoglobulin
PCR: polymerase chain reaction
SAG2: the surface antigen gene 2
